# Targeting mTOR in Acute Lymphoblastic Leukemia

**DOI:** 10.3390/cells8020190

**Published:** 2019-02-21

**Authors:** Carolina Simioni, Alberto M. Martelli, Giorgio Zauli, Elisabetta Melloni, Luca M. Neri

**Affiliations:** 1 Department of Medical Sciences, University of Ferrara, 44121 Ferrara, Italy; 2 Department of Biomedical and Neuromotor Sciences, University of Bologna, 40126 Bologna, Italy; alberto.martelli@unibo.it; 3 Department of Morphology, Surgery and Experimental Medicine, University of Ferrara, 44121 Ferrara, Italy; giorgio.zauli@unife.it (G.Z.); elisabetta.melloni@unife.it (E.M.); 4 LTTA-Electron Microscopy Center, University of Ferrara, 44121 Ferrara, Italy

**Keywords:** Acute Lymphoblastic leukemia, targeted therapy, mTOR, metabolism, cell signalling

## Abstract

Acute Lymphoblastic Leukemia (ALL) is an aggressive hematologic disorder and constitutes approximately 25% of cancer diagnoses among children and teenagers. Pediatric patients have a favourable prognosis, with 5-years overall survival rates near 90%, while adult ALL still correlates with poorer survival. However, during the past few decades, the therapeutic outcome of adult ALL was significantly ameliorated, mainly due to intensive pediatric-based protocols of chemotherapy. Mammalian (or mechanistic) target of rapamycin (mTOR) is a conserved serine/threonine kinase belonging to the phosphatidylinositol 3-kinase (PI3K)-related kinase family (PIKK) and resides in two distinct signalling complexes named mTORC1, involved in mRNA translation and protein synthesis and mTORC2 that controls cell survival and migration. Moreover, both complexes are remarkably involved in metabolism regulation. Growing evidence reports that mTOR dysregulation is related to metastatic potential, cell proliferation and angiogenesis and given that PI3K/Akt/mTOR network activation is often associated with poor prognosis and chemoresistance in ALL, there is a constant need to discover novel inhibitors for ALL treatment. Here, the current knowledge of mTOR signalling and the development of anti-mTOR compounds are documented, reporting the most relevant results from both preclinical and clinical studies in ALL that have contributed significantly into their efficacy or failure.

## 1. Introduction

Aberrant intracellular signalling pathways and inadequate continuous activation of cellular networks commonly result in abnormal growth and survival of malignant cells. The PI3K/protein kinase B (Akt)/mTOR network initiates and controls multiple cellular activities, including mRNA translation, cell cycle progression, gene transcription, inhibition of apoptosis and autophagy, as well as metabolism [1,2,3,4,5]. Constitutive activation of this pathway not only promotes uncontrolled production of malignant cells but also induces chemotherapy resistance mechanisms, also in leukemias. ALL is an aggressive malignancy of lymphoid progenitor cells in both pediatric and adult patients. In adults, 75% of cases develop from precursors of the B-cell lineage, the others consisting of malignant T-cell precursors [5,6,7,8,9,10]. T-ALL is also found in a range of 15% to 20% in children, affecting boys more than girls. Modern genomic approaches have identified a number of recurrent mutations that can be grouped into several different signalling pathways, including Notch, Jak/Stat, MAPK and PI3K/Akt/mTOR. Phosphatase and tensin homolog (PTEN), which acts as a tumour suppressor gene, represents one of the main negative regulator of PI3K/Akt/mTOR network. PTEN is the key regulator of phosphatidylinositol (3,4,5)-trisphosphate (PIP3) dephosphorylation into phosphatidylinositol (4,5)-bisphosphate (PIP2), thus blunting PI3K activity. In human T-ALL, PTEN is often mutated or deleted, leading to the upregulation of PI3K/Akt/mTOR, in combination with additional genetic anomalies [11,12]. Therefore, targeting the PI3K/Akt/mTOR signalling network has been investigated extensively in preclinical models of ALL, with initial studies focused on mTOR inhibition, demonstrating significant efficacy for mTOR drugs used as single inhibitors and synergistic effects in association with conventional chemotherapy [13]. 

It should be highlighted that, in addition to the standard chemotherapy, other treatment options such as immunotherapy represent today a new pharmacological approach, by targeting ALL surface markers expressed on B lymphoblasts, that are, CD19, CD20 or CD22 [14]. One immunotherapy strategy is represented by the bispecific T-cell engager (BiTE) antibodies, that bind the surface antigens on two different target cells, generating a physical link of a tumour cell to a T cell: from one side they can recognize the malignant B-cells through the CD19 and from the other side they activate T-cell receptor (TCR) through the interaction with the CD3 receptor on T-lymphocytes [15,16]. Blinatumomab is a first-in-class BiTE antibody and it is a bispecific CD19-directed CD3 T-cell mAb that has induced durable responses in patients with B-cell malignancies [17]. Blinatumomab has demonstrated important response rates in minimal residual disease (MRD) positive and relapsed or refractory B-ALL, both in adults and in children [16]. Another immunotherapeutic strategy in relapsed/refractory CD22^+^ ALL is represented by Inotuzumab ozogamicin, a novel mAb against CD22 conjugated to the toxin calicheamicin [18]. Another promising new therapy is the adoptive immunotherapy using chimeric antigen receptors (CARs) modified T cells, developed in recent years. CARs are artificial engineered receptors that can target specific cancer cell surface antigens, activates T cells and, moreover, enhances T-cell immune function [19,20]. The first constructs consisted of CAR T cells targeting CD19 marker and today different other antigens are under development. It has to be underlined that a CD19-directed genetically modified autologous T-cell immunotherapy, Kymriah (Tisagenlecleucel), has already been approved by FDA for patients up to 25 years of age with relapsed or refractory B-cell ALL [21]. In pediatric patients and young adults, treatment consisting of fludarabine and cyclophosphamide followed by a single infusion of Kymriah induced a significant (63%) Complete Remission (CR), negative for MRD with an acceptable benefit–risk profile for this patient population (see also www.clinicaltrials.gov: NCT02435849). 

However these immunotherapies are not considered completely curative and this is due to the fact that deadly relapses are common (median overall survival is <8 months with Blinatumomab and Inotuzumab ozogamicin and ~50% of CAR-T relapse within a year). Thus, novel approaches and further studies are needed.

Concerning mTOR inhibition, Rapamycin (Sirolimus) was first discovered as a new antifungal agent in soil samples from Rapa Nui, from which it received its name. In 1999 it was approved by FDA to prevent immune rejection of transplanted organs and studies in the budding yeast Saccharomyces cerevisiae revealed that a serine/threonine (Ser/Thr) protein kinase was the mediator of rapamycin toxic effects in yeast (Target of Rapamycin, TOR) [22]. mTOR is a 289 kDa serine/threonine kinase that works as a regulator of cellular progression and metabolic mechanisms in response to nutrients and hormonal signals [23]. mTOR forms with other components two distinct complexes, mTOR complex 1 (mTORC1) and mTOR complex 2 (mTORC2), that differ from each other according to their different functionality [24]. These complexes have in common the catalytic mTOR subunit and other three known complex components, mLST8, DEP domain-containing mTOR-interacting protein (DEPTOR) and Tti1/Tel2 [22,25,26,27]. mTORC1 is Rapamycin-sensitive and has unique components including regulatory-associated protein of mammalian target of rapamycin (raptor) and the 40 kDa proline-rich Akt substrate (PRAS40); mTORC2 comprises rapamycin insensitive companion of mTOR (rictor), mSIN1 and Proctor 1/2 as its specific components [28,29,30].

In this review we will summarize the current knowledge of mTOR signalling, the roles of its complexes, the mTOR involvement in the metabolism process, its relevance in ALL diseases and the status of mTOR inhibitors, reporting the most consistent results obtained from both preclinical and clinical analysis in ALL.

## 2. Activity of mTOR Complex 1

mTORC1 represents a nutrient/energy/redox sensor and is a controller of protein translation, cell metabolism and growth. For protein synthesis cells must have enough energy resources, nutrient support, oxygen abundance and proper growth factors to begin mRNA translation [31,32].

The binding of different growth factors and various cytokines to cell surface tyrosine kinase receptors leads to the PI3K/Akt/mTOR network activation. In particular, a key negative regulator of mTORC1 is TSC1/TSC2, a heterodimer with GTPase activity consisting of tuberous sclerosis 1 (TSC1) and tuberous sclerosis 2 (TSC2). This complex acts as a molecular switch for mTORC1 activity: during high stress situations, mTOR activity is blocked by this heterodimer, while it is again reactivated under favourable circumstances, that are, with growth factor stimulation and high cell growth conditions. The GTP-bound form of Ras homolog enriched in brain (Rheb) directly cooperates with mTORC1, strengthening its kinase activity and inducing proliferation and cell survival. As a Rheb GTPase-activating protein (GAP), TSC1/TSC2 negatively regulates mTORC1 by converting Rheb into its inactive form [33]. 

Among the upstream signalling networks, Akt and extracellular-signal-regulated kinase (ERK1/2) inactivate TSC1/2 and thus activate mTORC1.

Activated mTORC1 directly phosphorylates eIF4E-binding protein 1 (4E-BP1) and ribosomal S6 kinase (S6K), inducing protein synthesis [34,35,36]. The phosphorylation of 4E-BP1 impedes its binding to cap-binding protein eIF4E complex and leads to the initiation of cap-dependent translation. S6K controls the translation of several mRNAs that encode for ribosomal proteins and other constituents of the translational machinery such as elongation factors (i.e., the eukaryotic elongation factor 2 or eEF2). mTORC1-dependent anabolic induction is achieved through the phosphorylation of the downstream kinase S6K, as well as 4E-BP1, Lipin1 (lipid synthesis) and ATF4 (nucleotide synthesis). mTORC1 also up-regulates the glycolytic pathway by stimulating the transcriptional activation capability of Hypoxia Inducible Factor 1α (HIFα), a positive regulator of many glycolytic genes [37,38]. Furthermore, mTORC1 regulates the lysosomal function through transcription factor EB (TFEB), which controls the expression of many genes with key roles in lysosomal biogenesis and autophagy mechanism [39]. TFEB-mediated endocytosis induces the assembly of the mTORC1-containing nutrient sensing complex through the formation of endosomes, with further activation of Akt (Akt p-T308) [40].

mTORC1 promotes cell growth by blocking catabolic pathways such as autophagy, that represents the major degradation pathway in eukaryotic cells [41,42,43]. The inhibition of mTORC1 stimulates autophagy and this is correlated, for example during amino acid starvation condition, with the activation of the mTORC1 direct substrate Unc-51 like autophagy activating kinase (ULK1, including ULK2 isoform) [44]. The phosphorylation of ULK1 is a key signalling mechanism through which starvation-induced autophagy is regulated. Therefore, under starvation, activated phosphatases such as PP2A lead to a rapid dephosphorylation of ULK1, phosphorylated by mTORC1. ULK1 dephosphorylation correlates with an increment of the autophagic process [43]. Alterations in autophagy processes are correlated to different disorders including diabetes, cardiovascular disease, neurodegenerative diseases and cancer [45,46,47].

## 3. Activity of mTOR Complex 2

Differently to the role of mTORC1, little is known about the regulatory mechanism of mTORC2. mTORC2 specifically senses growth factors and controls cell survival, metabolism and actin rearrangement [48]. The nutrient-sensing role of mTOR is mainly dependent on mTORC1 [49]. mTORC2 is abnormally overexpressed in several cancer types and this characteristic leads to poor survival [50]. Although initial studies reported mTORC2 as a rapamycin-insensitive complex, there are actually several reports showing that rapamycin is capable of inhibiting mTORC2 upon longer exposure, most likely by negatively affecting the assembly of new mTORC2 complexes and therefore reducing mTORC2 levels that are usually required to maintain Akt/PKB signalling [51]. The different sensitivity to rapamycin may be explained by the fact that, in various cell types, a fraction of mTORC2 assembles with the FKBP12-rapamycin binding site not accessible to the drug. PTEN and FKBP12 expression represent possible modulators of rapamycin-mediated inhibition of Akt/PKB phosphorylation [51].

Liu and co-workers identified a correlation between PI3K growth factor stimulation and mTORC2 activity. SIN1 pleckstrin homology (PH) domain suppresses mTOR kinase domain function, as PIP3 interacts with the PH domain of mSIN1 to repress its inhibitory activity on mTOR, thus leading to mTORC2 activation [52].

mTORC2 substrates include the Ser/Thr cytosolic protein kinase Akt and protein kinase C (PKC), which share the hydrophobic motif at their phosphorylation site [53]. mTORC2 directly phosphorylates Akt at S473 residue and fully activates it [54,55]. Akt phosphorylation inhibits mTORC2 activity and thereby reduces the function of some Akt targets such as FoxO1/2 [56]. mTORC2 also stimulates serum and glucocorticoid-regulated kinase 1 (SGK1), a kinase that contributes to the regulation of glucose and creatine transporters, hormone release, inflammation, growth and apoptosis [57], belongs to the AGC family of protein kinases [54] and is involved in aberrant cell growth, survival and invasiveness. Nevertheless, recent findings have highlighted how mTORC2 could also act as a repressor of chaperone-mediated autophagy [58,59], which is frequently deregulated in numerous age-related disorders [60,61]. Moreover, mTORC2 increases Na^+^ transport and regulates cell migration [62,63]. 

**Figure 1:**
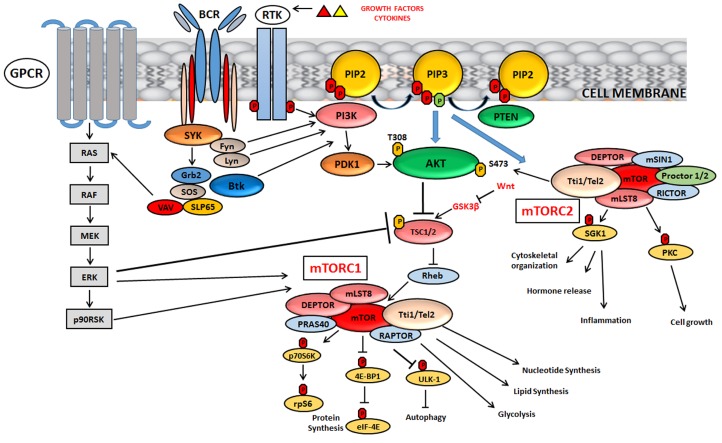
Signalling mechanisms and major functions of mTORC1 and mTORC2.

The major functions and downstream targets of mTORC1 and 2 are highlighted in Figure 1.

mTOR, activated by specific growth factors and cytokines, nutrients, cellular stress and oxygen levels, forms two distinct complexes, mTOR complex 1 (mTORC1) and mTOR complex 2 (mTORC2), that differ from each other according to their different activities. A key negative regulator of mTORC1 is TSC1/TSC2, consisting of tuberous sclerosis 1 (TSC1) and tuberous sclerosis 2 (TSC2). Among the upstream signalling networks, Akt and extracellular-signal-regulated kinase (ERK1/2) inactivate TSC1/2 and activate mTORC1. mTORC2 directly phosphorylates Akt at S473 residue leading to its complete activation. In B-cell development, PI3K/Akt/mTOR can also be activated by pre-BCR network, which involves constitutive activity of different kinases such as SYK, Fyn and Lyn. Wnt network, involved in embryonic development, can inhibit mTORC1 by blocking GSK3β expression. GSK3β is a positive modulator of the TSC complex. For other details, see the text.

## 4. mTOR Involvement in Metabolism

Metabolic modifications represent a hallmark of oncogenesis and tumour progression [64] and there is a growing interest in understanding these alterations associated with cancer cells resistant to therapy. Relapsed cancer cells, that have an aggressive phenotype, display an overexpression of the ATP-binding cassette transporter ABCB1 gene product and a high expression of the efflux pump P-glycoprotein (P-gp) [65]. The metabolic program often used by cancer cells is the aerobic glycolysis, which is characterized by increased glucose flux and production of lactate. Because of aerobic glycolysis tumour cells generate new lipids or amino acids necessary for cell proliferation [66]. Therefore, the metabolic alterations that characterize drug-resistance in cancer cells could represent an attractive therapeutic target. PI3K/Akt/mTOR network is a key regulator of glycometabolic homeostasis [67,68]. The active PI3K/Akt pathway is involved in glucose uptake by upregulating several cell surface glucose transporters (GLUT) expression and by stimulating several glycolytic enzymes, such as hexokinase [68]. mTORC1 activation is induced by different amino acids, particularly leucine and glutamine and also activates feedback mechanisms that further increase nutrient uptake to fuel anabolic reactions [69]. This complex is also involved in the upregulation of the expression of HIF1α, which has a key function in cell energy regulation, lipid and glutamine metabolic flux and promotes glycolysis with the conversion of glucose to pyruvate [70]. Moreover, mTORC1 positively regulates mitochondrial function and metabolism by selectively inducing translation of nucleus-encoded mitochondria-related mRNAs [71]. Upon starvation, mTORC1 activity is inhibited, alleviating the repression of ULK1/2. Active ULK1/2 phosphorylates key enzymes involved in the glycolytic pathway and promotes autophagy ensuring a good level of cellular energy and redox homeostasis [72]. Less is known about mTORC2 in growth factor signalling and metabolism. Recent data indicate that mTORC2 direct association with ribosomes ensures that this complex is active in cells that are growing and undergoing protein synthesis [63,68,73]. The metabolic profile of ALL is yet not well understood. However, it has been reported that in primary human peripheral T-ALL blood samples Glut1 and Hexokinase 1 and 2 (HK1 and HK2) were significantly elevated in comparison to T cells from healthy donors. In T-ALL, oncogenic Notch induced metabolic stress that stimulated 5′ AMP-activated kinase (AMPK). AMPK has growth suppressive functions and may act to block growth also in T-ALL [74]. Kishton et al demonstrated that unlike stimulated T cells, AMPK restrained aerobic glycolysis in T-ALL, also maintaining mitochondrial function to mitigate stress. The reduction of energy demand was modulated through mTORC1 inhibition-regulated anabolic growth, resulting in decreased aerobic glycolysis. The metabolic stress and apoptosis modulation by AMPK may thus provide new, potential approaches to treat T-ALL. To date, few studies have analysed the adaptations of the cellular cancer metabolism in a drug-resistance context. The transcriptional profile of several glucocorticoid-resistant T-ALL cells with high expression of genes related to the metabolic pathway was recently analysed. Treatment with rapamycin augmented cell sensibility to the glucocorticoid dexamethasone, indirectly correlating metabolism upregulation and mTOR function [75]. Compared with sensitive ALL cells, a recent study reported the metabolic profile of daunorubicin-resistant T-ALL cells and transcriptomic and metabolomic analysis revealed a higher dependence on glucose and a lower dependence on glutamine and fatty acids. The reduction of glutamate-ammonia ligase (GLUL) expression, the low levels of glutamine metabolism genes ASNS, ASS1, of the transporter SLC1A5 and the reduced level of pantothenic acid may reflect a more general adaptation based on metabolic rewiring that characterizes drug resistance in tumour cells [64]. Moreover, transforming oncogenes, such as RAS or BCR-ABL can impose significant metabolic requirements on glucose and energy supply [76,77]. Given the high presence of genetic lesions of B-lymphoid transcription factors in pre-B ALL cells, some studies analysed whether these transcription factors could restrict glucose and energy supply, thus getting in the way against malignant transformation. By combining ChIP-seq and RNA-seq studies, it was shown that two transcription factors, PAX5 and IKZF1, that are reported to have a key role in normal B-cell development, enforce a state of chronic energy deprivation and a constitutive activation of AMPK. In pre-B ALL patients, inducible wildtype reconstitution of PAX5 and IKZF1 decreased Akt activation, glucose-metabolism effectors and glucose transporters. On the other hand, a strong induced expression of glucose transport inhibitors was shown. NR3C1 (glucocorticoid receptor), TXNIP (glucose feedback sensor) and CNR2 (cannabinoid receptor) were identified as central effectors of B-lymphoid glucose restriction and energy supply. Indeed, the genetic loss-of-function of NR3C1, TXNIP and CNR2 significantly improved glucose uptake and increased ATP-levels [78]. Therefore, B-lymphoid transcription factors can represent metabolic gatekeepers limiting the amount of cellular ATP to levels insufficient to achieve malignant transformation. Moreover, the effectors NR3C1, TXNIP and CNR2 could represent valuable therapeutic targets for the treatment of pre-B ALL.

## 5. Targeted Therapy: Inhibition of mTOR in ALL

The crucial function of mTOR as a regulator of Akt, its involvement in modulation of other signalling cascades such as NOTCH-1 (principally via-mTORC2) and its ability in monitoring metabolic functions and energy homeostasis in healthy and tumoral cells led to a growing interest in developing targeted and personalized ALL therapies. There is a marked interest in targeting mTOR protein kinase, for cancer therapy, also based on genetic studies showing selective effects on tumour cells following mTOR inactivation [79]. Indeed, some recognized molecular lesions related to adverse clinical prognosis in ALL are involved in mTOR-mediated signalling. Based on these observations, three classes of mTOR inhibitors are included in the scenario of ALL treatment: allosteric inhibitors [(rapamycin and rapalogs, that are, RAD001 (everolimus), CCI-779 (temsirolimus)] mainly targeting mTORC1 [80,81,82], ATP-competitive dual PI3K/mTOR inhibitors [6,83,84] and mTOR kinase inhibitors (TORKIs) that specifically have as a target mTORC1 and mTORC2 but not PI3K [79,83,85]. Rapamycin and rapalogs belong to the mTOR first generation inhibitors, are the most well documented drugs and have shown antitumor activity in clinical trials, not only for ALL cases but also in non-haematological neoplasms. Rapamycin interacts with the intracellular receptor, FK506-binding protein 12 (FKBP12) and interferes with growth-stimulating cytokine signalling [86]. Together with the immunophilin FKBP12, rapamycin/rapalogs associate with the FKBP12-rapamycin-binding (FRB) domain of mTORC1 [87]. This association results in decreased interactions between mTOR and Raptor with a consequent downregulation of mTORC1 activity [88]. This was reported to occur by steric hindrance through reduction in the size of the mTOR active-site [87]. Rapamycin has been tested alone and in combination with Janus Kinase (JAK), ABL protein inhibitors [84], focal adhesion kinase (FAK) or also with cyclin D3 (CCND3) and CDK4/6 inhibitors [89,90] in xenografts model and in in vitro cancer cell lines, showing relevant synergistic effects and induction of cellular mechanisms as autophagy. Nevertheless, rapamycin has also been reported to have pharmacological limitations. For this reason rapamycin derivatives (so called “rapalogs”) for the treatment of ALL have been developed, with minor immunosuppressive activity [86] and greater antitumoral action. Among these rapalogs, RAD001 has been widely investigated in in vivo and in vitro models for its antiproliferative activities. It is more selective for the mTORC1 protein complex, with lower impact on mTORC2 and different trials are underway also in solid tumours like gastric cancer and hepatocellular carcinoma. Numerous studies reported the efficacy of RAD001 in inducing caspase-independent cell death and cell cycle regulation changes [91] or in overcoming resistance in ALL versus several inhibitors like Tirosine Kinase Inhibitors (TKI) [92]. However, limited apoptosis was reported by rapalogs, despite the delayed progression of tumour growth. Therefore, second-generation anti mTOR drugs were developed, to compete with the catalytic site of mTOR blocking the feedback activation of PI3K/Akt network (mTOR kinase inhibitors) and to repress both mTOR complexes, overcoming some of the limitations of rapalogs. BEZ235 and BGT226, in addition to others that will be described in the next sections, belong to the PI3K/mTOR inhibition class and for both are reported significant apoptotic and anti-leukemic activity in vitro and in vivo [8,93,94,95]. AZD8055, AZD2014, TAK-228, CC-223 and OSI-027 are examples of TORKIs and entered phase 1/2 clinical trials for the treatment of different solid tumours but not yet for ALL. mTOR inhibition also appears to be effective when combined with conventional chemotherapy in ALL [85,96] and with drugs targeting the epigenetic machinery, inducing apoptosis, as well as increased mitochondria sensitivity to initiate cell death [97] and inhibiting BCL2 protein family leading to increased cytotoxicity [98]. 

mTOR inhibition either alone or in association with conventional ALL therapies or with targeted drugs for different cellular cascades is able to block distinct mechanisms of cell survival in ALL, providing a strong rationale for the investigation of mTOR inhibition particularly in the setting of resistance to chemotherapeutic drugs. In the next sections the most recent advances in mTOR inhibition in B- and T-ALL are discussed, pointing out the therapeutic importance of this protein kinase in this hematologic malignancy treatment.

### 5.1. mTOR Inhibition in T-ALL

T-ALL is a neoplasm caused by numerous and relevant genomic lesions that affect the development of T cells [99,100,101]. In T-ALL, high expression of mTOR was reported to be more frequent in adults than in children [102]. Literature data documented, in several preclinical studies, a good efficacy of allosteric mTOR inhibitors in T-ALL cells, with cytostatic effects [103,104]. Rapamycin and CCI-779 are able to block Interleukin-7 (IL-7)-dependent T-ALL growth [105]. Indeed, the two inhibitors induced T-ALL cell death when cultured in the presence of this interleukin. Moreover, in T-ALL cell lines, rapamycin re-establishes the expression of p14, p15 and p57 genes, that normally act as cell cycle regulators and are reported to be inactivated in adult ALL through promoter methylation [90]. Demethylation of the promoter of the G1/S transition genes mediated these effects, accompanied by a marked decrease of mTOR and p70S6K expression. However, the molecular mechanism concerning this regulation still has to be clarified. Rapamycin and derivatives could be significantly combined with drugs currently employed in the treatment of T-ALL, that is, Doxorubicin (Doxo) [89,105], cyclophosphamide [106] and methotrexate [107]. Pikman et al. documented also a good synergism in the combinations of the CDK4/6 inhibitor LEE-01 (ribociclib) with the rapalog RAD001 and with glucocorticoids (GCs), currently used in the therapeutic protocols in the treatment of T- and B-ALL. The efficacy of the combination rather than the single agent was shown both in vitro and in T-ALL mouse models [89]. Other relevant allosteric mTOR inhibitors combinations to be cited are those with inhibitors of NOTCH1 signalling network. This evolutionally conserved signalling network, with key roles in modulation of haematopoiesis, cell growth, apoptosis and angiogenesis, is commonly dysregulated in T-ALL representing the most common abnormality in this subtype. NOTCH1 can activate PI3K/AKT/mTOR network at multiple levels, regulating cell size, glucose accumulation and glycolysis during T-cell development [108]. Consequently, inhibition of NOTCH1 correlates with suppression of mTOR, highlighting the close interconnection between the two signalling cascades. Different PI3K upstream signalling receptors, such as the interleukin 7 receptor α chain (IL7RA), are upregulated by NOTCH1 signalling in T-cell progenitors. The oncogene MYC, prominent target gene in T-cell transformation, is able to revert the inhibitory effects of blocking NOTCH1 on the mTOR network [108,109,110]. The dual inhibitor PI3K/mTOR inhibitor PKI-587 (Gedatolisib) exhibited a significant inhibitory effect on T-ALL leukemia cells and in T-ALL patients with poor prognosis. In T-ALL cells PKI-587 blocked proliferation and colony formation and, in immune-deficient mouse models, delayed tumour progression, enhancing the survival rate. PKI-587 was also particularly effective in CRLF2/JAK-mutant models with a 92.2% leukemia proliferation reduction versus vehicle controls and with significantly prolonged mice survival [83]. Preclinical studies of the dual PI3K/mTOR inhibitor BEZ235 showed anti-proliferative activity in ALL cell lines [111,112]. In particular, this drug induced anti-leukemic activity when associated with glucocorticoids in vitro and in vivo models [112,113]. In PTEN null cells, BEZ235 controlled GC-resistance by increasing the level of the proapoptotic BIM protein, inducing a marked apoptosis [112,114]. The pronounced antiproliferative effects of BEZ235 have also been observed in Jurkat and MOLT4 T-ALL cells, when administered with cytarabine (AraC), Doxo or glucocorticoids [111]. TORKIs interfere only with the mTOR catalytic domain [54]. The levels and activation status of regulators of mTORC1 were recently examined in γ secretase inhibitors (GSI)-resistant T-ALL cells. These inhibitors are reported to suppress Aβ peptide and to suppress the Notch signalling pathway [115]. The combination of the mTOR inhibitor AZD8055 with the BH3-mimetic, ABT-263, induced a decreased phosphorylation of 4EBP1 and S6, also lowering MCL-1 expression and inducing tumour regression in vivo [116]. In T-ALL Jurkat cells, the mTOR kinase inhibitor OSI-027 induced c-Myc reduction and activation of the PUMA BCL2 family member. At the same time, the mTORC2 activity inhibition, with 4EBP1 phosphorylation, resulted in NF κB–mediated expression of the early growth response 1 (EGR1) gene, which encodes for the proapoptotic protein BIM. Therefore, different signalling networks are involved in T-ALL apoptosis, after OSI-027 treatment [117]. Table 1 reports a summary of the main mTOR inhibitors in T-ALL models. However, there are still few data concerning the efficacy of this class of inhibitors for T-ALL, while some Phase I clinical studies conducted mostly on solid tumours have been released [118]. Therefore, further studies are needed to predict valuable therapeutic protocols with TORKIs in T-ALL models.

### 5.2. mTOR Inhibition in B-ALL

B-ALL is characterized by the uncontrolled proliferation of B-cell precursors [119] and is further classified by the differentiation status as pro-B, common, precursor B (pre-B) and mature B-cell ALL. Pro-B ALL is an ALL unfavourable subset in childhood and adults and lacks the B cell marker of therapeutic resistance CD10 [120]. Pre-B ALL represents the most common type in adults, with cells that characteristically co-express CD10 and CD19 surface markers [121], while mature B cell ALL is sometimes called Burkitt type ALL because it shares similar characteristics to the Burkitt lymphoma [122]. Despite considerable progress in treatment protocols, B-ALL displays a poor prognosis in about 15–20% of pediatric cases and about 60% of adult patients. Factors for higher risk of relapse in adults include the Philadelphia chromosome alteration (Ph) formed upon the t(9;22) reciprocal chromosomal translocation, with consequent formation of a Bcr-Abl chimera gene [107]. The incidence of Ph^+^ B-ALL increases with age and occurs up to 50% of B-ALL diagnosed in subjects over 50 years old [85]. Philadelphia (Ph)-like ALL is a recently characterized subtype. This subgroup has been reported to have a high expression of cytokine receptors and signalling tyrosine kinases, resulting in kinase activation through stimulation of ABL and JAK/STAT pathways. This subtype is very aggressive and the gene expression profile is reported similar to that of Ph^+^ B-ALL, although the Bcr-Abl fusion protein is lacking. For this aspect, the development of sensitive, cost-effective and commercially available diagnostic approaches are needed in order to better identify this type of patients [123]. Many Small Molecule Inhibitors (SMI) have been or are in clinical development for both T- and B-ALL subtype [10,124,125,126,127]. Indeed, hyperactivation of PI3K/Akt/mTOR network is usually detected also in B-ALL subtype [128] and correlates with poor prognosis and drug resistance both in adults and in pediatric B-ALL patients [129,130]. In pre-B ALL the combination of RAD001 with the Akt inhibitor MK-2206 significantly exerts anti-leukemic activity, with increased apoptosis and autophagy induction [5]. For this ALL subtype and in particular for Ph^+^ B-ALL, mTOR inhibitors are usually given in combination with other signalling cascade drugs or in association with TKI, since the antiproliferative activity of a single mTOR inhibitor is not often effective in overcoming drug-resistance.

### 5.3. mTOR Activity in B-ALL

High basal levels of Akt and mTOR activation in B cell leukemias and lymphomas have been reported in different works [10,131,132]. Recent studies in immune cells highlighted that mTOR not only couples nutrient availability with cell proliferation but also controls cell differentiation and activation-induced responses in B and T lymphocytes, natural killer cells, neutrophils, macrophages and other cellular types [132]. By assessing the total expression of mTORC1 signalling proteins Raptor and mTOR, as well as the phosphorylation of mTORC1 downstream S6 ribosomal (S6R) and 4EBP1 proteins, it has been found that mTORC1 pathway is predominantly activated during the pro-B, large and small pre-B cells stages and to a lesser extent in resting immature and mature B cells in the bone marrow. These results are consistent with the expression of the IL-7 receptor (IL-7R) during the pro-B and pre-B cell processes and the activation of mTORC1 downstream of IL7R. The importance of mTOR in cell maturation and differentiation has been documented in a study involving an hypomorphic mouse, characterized by a neomycin cassette insertion in an intron that partially disrupted mTOR transcription. A partial block in B-cell development in the bone marrow was detected with a reduction of pro-B, pre-B and immature B cell populations, simultaneously with a reduced mature B cell populations in the spleen. mTORC1 signalling is therefore specifically required in early B cell development [133,134]. mTORC2 signalling is also important in B cell development and function. Indeed, Rictor knockout mice reported a decrease in the quantity of mature B cells in the peripheral blood and in the spleen and impaired early B cell development in the bone marrow. Rictor deficient B cells exhibited an aberrant increase in FoxO1 and recombination-activating gene 1 (Rag-1) and rapamycin aggravated the Rictor deletion-induced defect in B cells via the inhibition of mTORC1 activity [135]. The mTOR hyperactivation in B-ALL is strictly dependent on oncogenic drivers, such as BCR-ABL1 fusion gene, on kinases commonly found aberrant in B-ALL such as cytokine receptor-like factor 2 (CRLF2) or JAKs, on hyperactivated growth receptors as colony-stimulating factor 1 receptor (CSF1R) and on overexpressed fms-related tyrosine kinase 3 (FLT3) and IL7R. Besides BCR-ABL1 fusion gene, the ETV6/RUNX1 (E/R) mutation is frequently found in childhood B-ALL and literature reported the importance of using dual PI3K/mTOR drugs for a marked inhibition of cells harbouring E/R mutation and a decreased cell resistance to glucocorticoids [136]. Activating mutations in upstream kinases such as protein kinase C delta (PKCδ) or the cytoplasmic protein spleen tyrosine kinase (SYC), are the most common causes of mTOR hyperactivity. SYK, which is downstream of pre-BCR signalling, promotes the activation of PI3K/Akt pathway [137,138]. Concerning Ph^−^ B-ALL cases, literature reported that the upregulation of the PI3K/Akt/mTOR network could be dependent on constitutively active pre-BCR signalling that characterizes approximately 13% of Ph^−^ B-ALL cases [139]. Rapamycin, RAD001 and CCI-779 have all been tested in various clinical trials for ALL in combination with multi-agent chemotherapy, with promising results including CR of relapsed childhood ALL [140]. However, a disadvantage of rapalog therapy is the upregulation of the pro-survival/proliferation PI3K pathway, which occurs through loss of mTORC1 negative feedback on PI3K and on mTORC2 and the incomplete suppression of the mTORC1 substrate 4E-BP1 [140]. Moreover, rapamycin and rapalogs suppress p70S6K1 phosphorylation and switch off the Insulin receptor substrate (IRS)-dependent negative feedback mechanism that prevents aberrant activation of PI3K/Akt network in response to insulin/IGF-1, leading to Akt/mTORC1 activity up-regulation [54]. Treatments with TORKIs more effectively block mTORC1 substrate phosphorylation relative to rapalogs and also inhibit mTORC2 activity, thus attenuating the Akt pathway and reducing the unwanted upregulation of PI3K pathway. Different pharmacological combinations involving mTOR inhibitors, dual PI3K/mTOR inhibitors and other drugs are used to overcome possible other resistance mechanisms such as the upregulation of the Ras/MAPK/ERK pathway or Receptor Tyrosine Kinase (RTK) overexpression. Targeting BCL-2 proteins represents a direct approach for apoptosis subsequently to mTOR inhibition, therefore modulating the anti-apoptotic components (BCL-2 or BCL-XL) and proapoptotic sensors (BAD or PUMA), activators (BIM) or effectors (BAX) [140]. Analysis of structurally distinct TORKIs in B-ALL reported that mTOR inhibition was capable to induce apoptosis when compared to rapamycin [141,142]. The main challenge will be to achieve the most advantageous drug combinations targeting multiple key survival pathways, at the same time selective for cancer cells but with little or no side toxicity that at present constitutes a major concern.

#### 5.3.1. Targeting mTOR in Ph^+^ B-ALL and in Ph-like B-ALL

The discovery of TKIs has led to significant improvement in the treatment of Chronic Myeloid Leukemia (CML) and Ph^+^ ALL [143,144,145,146]. However, despite the implementation of TKI for the treatment of Ph^+^ B-ALL, survival outcomes still remain poor compared to Ph^−^B-ALL [124]. The combination of Imatinib (IM, first-generation TKI) with standard chemotherapy or with allogeneic hematopoietic stem cell transplantation (HSCT) has significantly ameliorated the survival of Ph+ ALL [147,148,149]. More recently, second- or third-generation TKI (dasatinib, nilotinib, bosutinib and ponatinib) have been used as first-line treatment in Ph^+^ B-ALL with positive outcomes [150,151]. In Ph^+^ B-ALL, the BCR-ABL fusion gene directly activates the mTOR network, that could represent a mechanism of disease resistance to TKI therapy [152]. More than 50 types of mutations in the BCR–ABL fusion gene have been identified including Y253H, E255K, M351T, G250E and T315I [146]. Targeting the signalling pathway downstream from BCR-ABL, rapamycin could circumvent imatinib resistance in cells carrying the T315I mutation. This mutation usually confers resistance to all first-and second-generation TKIs, except to the third-generation TKI Ponatinib (AP24534), that represents the treatment of choice for CML [146,153] and Ph^+^ ALL [154,155]. Ponatinib has also potent activity against FLT3, that confers resistance to imatinib, nilotinib and dasatinib [156]. The efficacy of ponatinib versus imatinib is actually in clinical studies for Ph^+^ ALL, besides another study focused on the effects of ponatinib with the monoclonal antibody Blinatumomab in Ph^+^ and Ph^−^ ALL (see www.clinicaltrials.gov: NCT03263572). Martinelli et al. published a study revealing a promising activity of ponatinib in patients affected by CML and positive for T315I [157]. Its efficacy is better documented in association with other molecules, such as blinatumomab and the WEE1 inhibitor AZD-1775, in patients with relapsed/refractory Ph^+^ disease and in T-ALL cell lines [158,159,160]. Imatinib could cause an abnormal activation of the mTOR pathway, leading to treatment resistance. The addition of rapamycin to imatinib mesylate overcame this effect in Ph^+^ B-ALL and induced apoptosis [161]. Moreover, rapamycin could potentiate the proliferation inhibition induced by daunorubicin in Ph^+^ B-ALL cells and primary samples and at the same time eliminated the abnormal effect of daunorubicin to aberrantly upregulate mTORC1 signalling [162]. Therefore, there is a rationale also in using mTOR inhibitors for this ALL subtype and this may be a mechanism of improving outcomes in Ph^+^ B-ALL. The importance and efficacy of the co-treatment of allosteric mTOR inhibitors with conventional chemotherapy or with TKI therapy has also been highlighted by Kuwatsuka et al., whose study demonstrated that RAD001 overcame resistance to imatinib by targeting in vitro and in vivo a mostly quiescent Ph^+^ B-ALL cell subset (CD34^+^/CD38^−^) [75]. RAD001/imatinib co-treatment induced in vitro apoptosis of CD34^+^/CD38^−^ cells more selectively than RAD001 alone and the treatment was more effective in reducing Mcl-1 expression than either drug alone. Co-treatment with RAD001 can therefore overcome resistance to imatinib in Ph^+^ B-ALL leukemic stem cells (LSCs), introducing more effective therapeutic treatments aimed to lower the number of patients who relapse after TKI treatment. PI-103, BEZ235 and PKI-587 (Gedatolisib) have also been employed in pre-clinical models of B-ALL subtypes [10]. PI-103 was more effective than rapamycin in suppressing proliferation of Ph^+^ B-ALL leukemia cells treated with imatinib [148], both in mouse pre-B ALL and human CD19^+^ CD34^+^ Ph^+^ ALL cells. BEZ235 was reported to induce apoptosis in Ph^+^ B-ALL nilotinib-resistant cells, leading to a marked downregulation of the anti-apoptotic MDM2 protein (or human homolog of the murine double minute-2) [163]. As for the dual PI3K/mTOR inhibitor PKI-587 (Gedatolisib), it displayed antitumoral activity in childhood B-ALL patient-derived xenograft models having various Ph-like genomic alterations [84]. Regarding TORKIs, actually only PP-242 and MLN0128 (known also as INK128) have been tested in Ph^+^ ALL models. PP242 displayed more significant cytotoxic effects and a more complete inhibition of mTORC1 in combination with Imatinib in Ph+ SUP-B15 cells, with a marked up-regulation of the apoptosis associated proteins (Bax and cleaved caspase-3) [150]. In preclinical models of paediatric and adult Ph^+^ B-ALL but also in Ph^−^ B-ALL, MLN0128 suppressed proliferation and increased the efficacy of the second generation TKI Dasatinib, supporting the hypothesis for potential clinical analysis of this TORKI. Moreover, in in vivo models, this inhibitor displayed a low toxicity [164]. Further studies are needed to highlight the importance of TORKIs for a more precise and personalized treatment in Ph^+^ B-ALL models, with protocols that also involve more than one inhibitor targeting different signalling cascades. Beagle et al. demonstrated, for example, the benefit of using histone deacetylases inhibitors (HDACi) in combination with TORKIs. Indeed, in Ph^+^ and Ph^−^ B-ALL and in primary pediatric B-ALL, the cytotoxic role of TORKIs can be augmented by the HDACi vorinostat or panobinostat, with a resulting increased expression of pro-death genes and transcription factors [97]. The high risk pediatric Ph-like B-ALL cohort, that suffers high rates of relapse and mortality, frequently displays a panel of genetic rearrangements in the cytokine receptor like factor 2 (CLRF2), JAK 1/2/3, IL-7R, FLT3 or platelet-derived growth factor receptor-β (PDGFRB) [165]. All of these aberrations have the potential to modulate the PI3K/Akt/mTOR network leading to its aberrant activation. It has been shown that, in xenograft models with and without CRLF2 and JAK genetic lesions, rapamycin reduced leukemia blasts, prolonging also survival [166]. Gotesman et al. further gave relevance to mTORC1 inhibition combining TORKIs with dasatinib in ABL-rearranged Ph-like B-ALL. The combination was more effective than TKI alone against patient-derived Ph-like B-ALL cells, suggesting a rationale for clinical testing of TKI associations with TORKIs in pediatric and adults Ph-like B-ALL patients and new therapeutic strategies in this poor prognosis subtype of B-ALL [143]. 

#### 5.3.2. Targeting mTOR in Ph^−^ B-ALL

Evidence suggests that B-cell receptor (BCR) plays an important role not only in Ph^+^ B-ALL but also in Ph^−^ B-ALL, where several molecules, such as IL-7, modulate survival and cell death mechanisms [167]. Indeed, the precursor-B-cell receptor (pre-BCR) activation depends on different signals that are required to initiate several aberrant cellular processes in pre-B cells, such as abnormal proliferation. Prior to become a functional mature B cell, B cell progenitors must successfully proceed through several checkpoints that ensure in mature B cells the expression of functional immunoglobulin receptors capable of recognizing a wide-array of antigens [132]. Pre-BCR receptor ensures pre-B cells differentiation into mature B-cells. Therefore, pre-BCR acts as a checkpoint in B-cell development and is involved in the recombination of light chain gene IgL through the termination of surrogate light chains (SLC) expression [168]. Together with BCR, mature B cells development is strictly correlated to the activation of the receptor for the tumour necrosis factor (TNF) family cytokine, BAFF, that signals mainly through NF-κB pathway [169]. In Ph^+^ALL, the oncogenic fusion BCR-ABL stimulates some pre-BCR downstream effectors, such as Bruton’s tyrosine kinase (BTK), the transcription regulator protein BACH2 and B Cell CLL/Lymphoma 6 (BCL6) [138,170]. In Ph-B-ALL the number of cases displaying high expression of BCL6 protein and a constitutively active BCR signalling is around 13% [171]. Several studies reported the efficacy of different pharmacological combinations in inducing downregulation of PI3K/Akt signalling, as an arm of BCR pathway. In Pre-BCR^+^ ALL cells the BTK inhibitor ibrutinib induced the suppression of some pre-BCR signalling negative regulators, inhibited the phosphorylation of PI3K/Akt network and its substrates, reduced BCL6 levels and synergized with glucocorticoids. Moreover, it induced apoptosis and prolonged survival in Pre-BCR^+^ ALL mouse models [172]. On a panel of B-ALL, the Akt inhibition and the reduction of phosphorylation of its downstream target glycogen synthase kinase 3β (GSK3β) have been detected after treatment for 48h with the PKCβ selective inhibitor enzastaurin (ENZ). PKCβ represents a key mediator of BCR and pre-BCR signalling. The reduction of activated GSK3β correlated with an abnormal accumulation of β-catenin [173]. β-catenin, that belongs to the Wnt/β-catenin signalling, is involved in stem cell abilities to self-renew and is implicated in growth and drug resistance of B-ALL cells. Wnt was reported to inhibit mTORC1 by inhibiting GSK3β, positive regulator of the TSC complex [132]. In B-cell precursor (BCP) ALL with the TCF3-PBX1 (E2A-PBX1) gene fusion, the PI3K delta (p110δ) inhibitor idelalisib represents a promising pharmacological approach for this subtype, among a panel of 302 investigational and approved anti-neoplastic drugs. The idelalisib insensitive 697 BCP-ALL cell line harbours an activating NRAS mutation, which may cause resistance to p110δ inhibition [174]. Also an aberrant expression of C-X-C chemokine receptor type 4 (CXCR4), may influence additional drug sensitivity of this cell subtype. Concerning mTOR inhibition, Rapamycin, CCI-779 and RAD001 have been used also in pre-clinical models of Ph^−^ B-ALL. Rapamycin induced apoptosis and exerted anti leukemic effects in the pre-B ALL cell line T309 and the treatment, in vivo, with the mTOR inhibitor in transgenic mice displayed a reduction in nodal masses and a prolonged survival. Interestingly, the inhibitory effects of rapamycin could be reversed by IL-7. This suggests an important role of this cytokine in the control of mTOR activity in B-ALL cells [175]. Moreover, rapamycin has been reported to be synergistic with focal adhesion kinase (FAK) down-regulation in REH cells with significant down-regulation of cell growth, cell cycle and apoptosis [7]. CCI-779 significantly decreased survival and induced apoptosis of lymphoblasts from Ph^−^ B-ALL adult patients co-cultured with bone marrow stromal cells. This drug was also effective in vivo in a NOD/SCID xenograft model, where inhibition of mTORC1 showed a significant reduction in peripheral-blood blasts and splenomegaly [81]. Also RAD001 has proven its efficacy, especially in models of Ph^−^ pediatric B-ALL. RAD001 is able to synergize with conventional chemotherapy (i.e., vincristine) or novel agents (i.e., bortezomib) both in vitro and in vivo, with increased caspase-dependent but p53-independent cell killing [176,177]. In further several studies it has also been shown that this inhibitor induces autophagy [178] and caspase-independent programmed cell death [179]. Our group recently reported the synergistic effect of RAD001, in both Ph^−^ B-ALL cell lines and patient samples, with MK-2206, a specific, potent and orally bioavailable allosteric Akt inhibitor that targets both its catalytic and PH domains [180]. BEZ235 has also shown to exert anti-proliferative activity in Ph^−^ B-ALL cells [95], as well as the PI3K/mTOR inhibitor BTG226, that showed a more powerful effect than BEZ235 [95]. Both drugs, however, inhibited the proliferation of Ph^−^ B-ALL cell lines with a three log greater potency than RAD001 alone. It has also been recently reported that BEZ235 synergizes with the Bcl-2 inhibitor, GX15-070 (Obatoclax), in Ph^+^ and Ph^−^ primary B-ALL cells [181], representing a potent approach to counteract growth and survival of ALL cells. Regarding TORKIs, our group has recently documented that Torin-2, displayed cytotoxicity to a panel of Ph^−^ B-ALL cell lines and it was found that the drug as a single agent was able to suppress feedback activation of PI3K/Akt, whereas RAD001 needed the addition of MK-2206 to show the same effect [142]. Table 2 reports a summary of the main mTOR inhibitors in B-ALL models. An effective target inhibition coupled with safety characterization of the targeted drugs could help to better identify therapeutic responses with limited adverse side effects.

## 6. Clinical Trials

Actually some clinical trials are performed in both T- and B-ALL, some of them in completed status with results, others in recruiting phase. Among the different clinical trials, a Phase I/II study reported the results of RAD001 or CCI-779 in combination with chemotherapy for treatment of ALL patients with relapse episodes. The combination of RAD001 with Hyper-CVAD (Hyperfractionated Cyclophosphamide, Vincristine, Doxo and Dexamethasone) high-intensity chemotherapy in B-lineage or T-lineage acute leukemia patients significantly inhibited the phosphorylation of S6RP. Of note, the combination of Hyper-CVAD and RAD001 did not induce a relevant increased toxicity, compared with Hyper-CVAD alone (see www.clinicaltrials.gov: NCT00968253) [96]. In an early Phase I pilot study also rapamycin was given in combination with Hyper-CVAD in adults with B- and T-ALL and other aggressive lymphoid malignancies, with the aim to assess the feasibility, safety and toxicity of the drug combination (see www.clinicaltrials.gov: NCT01614197). Rapamycin efficacy is also under evaluation in combination chemotherapy with or without Donor Stem Cell Transplant in adult patients affected by Ph^+^ B-ALL (see www.clinicaltrials.gov: NCT00792948). The toxicity of CCI-779 in combination with dexamethasone, cyclophosphamide and etoposide is also under evaluation in children with relapsed ALL and in patients affected by lymphoma. In this clinical trial (see www.clinicaltrials.gov: NCT01614197) different analytical tests aimed on dose limiting toxicity (DLT) measurement or on rate of remission assessment will be performed. MRD and in general CCI-779 effect on glucocorticoid resistance and on the mTOR inhibition will be also evaluated. In Another Phase I trial CCI-779 was tested in combination with UKALL R3 re-induction protocol. Sixteen T- or B-ALL patients participated in the study. Unfortunately, the addition of CCI-779 to reinduction chemotherapy resulted in consistent toxicity, without disease reduction. (see www.clinicaltrials.gov: NCT01403415) [182]. In a third Phase I trial study, the activity of RAD001 was evaluated in combination with different drugs and conventional chemotherapy in childhood ALL having a first relapse at bone marrow level (see www.clinicaltrials.gov: NCT01523977) [183]. More than 20 patients were involved in the study. The drug combination was well tolerated and correlated with low level of MRD re-induction, thus enhancing further evaluations in the clinic for this combination efficacy. Further trials are ongoing for the evaluation of rapalogs efficacy (see for example www.clinicaltrials.gov: NCT03328104). BEZ235 is also under clinical evaluation, especially in patients with relapsed ALL. This study is currently in active status and the main objective is the identification of the patient MTD and BEZ235 DLT when administered twice daily. Moreover, changes in PI3K/Akt/mTOR network molecules in bone marrow are being analysed (see for example www.clinicaltrials.gov: NCT01756118). The selection of effective chemotherapy drugs that, in combination with targeted mTOR SMI, could be quite well tolerated with minimal toxicity is a priority for ALL treatment, with the aim to define accurate pharmacological protocols and to optimize the drug doses to alleviate adverse effects.

## 7. Conclusions

The mTOR signalling pathway is physiologically upregulated in different cellular mechanisms and can be aberrantly activated in human pathologies and in particular in tumours. The key role of mTOR in the early stages of leukemia and cell drug resistance has been documented in many scientific works [6,24,184]. 

The field of mTOR targeted therapies has reported a rapid progression over the past few decades and several mTOR network inhibitors have been analysed, with their own inhibitory activities and profiles on their levels of toxicity. However, the advances in knowledge on mTOR drugs is unfortunately still quite limited. Therefore, the challenge ahead is to understand the most suitable inhibitor that could exert a considerable efficacy with minor toxicity in each patient affected by cancer, in this case by ALL. Modern techniques such as kinase activity profiling [185] or next-generation sequencing analysis [186] will help to enter more specifically in the molecular aspects of signal transduction, pointing out the most appropriate druggable molecules or gene mutations and therefore to define the percentage of ALL patients that can benefit from drug-sensitiveness or drug-resistance. A better overview of the inhibitory and anti-proliferative effects of targeted inhibitors and their role on the complexity and diversity of the immune context of the tumour microenvironment could further improve therapy response [187]. 

The advances in the comprehension of the biological activities and of the impact that mTOR performs in ALL could give a significant contribution to the advancement of new therapeutic strategies, aimed to inhibit mTOR, for ameliorating ALL patient outcomes.

## Figures and Tables

**Table 1:** mTOR inhibitors used alone or in association in T-ALL.

Drug	Drug Target	Reported Synergism	Clinical Trials	Reference(s)
Rapamycin (Sirolimus)	mTORC1	Doxorubicin Janus kinase inhibitor ABL protein inhibitors Focal Adhesion Kinase (FAK) inhibitor Cyclin D3 and CDK4/6 inhibitors Cyclophosphamide Methotrexate γ-secretase inhibitors CCI-779	NCT00968253 NCT01184885	[80,82,84,86,87,88,89,90,96,105,106,107]
CCI-779 (Temsirolimus)	mTORC1	Doxorubicin	NCT01614197 NCT01403415	[80,81,82,96,105,142,182]
RAD001 (Everolimus)	mTORC1	LEE-01 Glucocorticoids	NCT03328104	[80,82,89,91,92,96,97,182]
PKI-587 (Gedatolisib)	PI3K/mTOR	-	-	[6,83,84]
BEZ235	PI3K/mTOR	cytarabine (AraC) Doxorubicin Dexamethasone	-	[6,84,111,112,113,114]
AZD8055	mTORC1/mTORC2	PP-242	-	[79,85,115]
OSI-027	mTORC1/mTORC2	-	-	[79,85,116]

**Table 2:** mTOR inhibitors used alone or in association in B-ALL.

Drug/Cells	Drug Target	Reported Synergism	Clinical Trials	Reference(s)
Rapamycin (Sirolimus) (Ph^+^ B-ALL, Ph^−^ B-ALL, Ph-like B-ALL)	mTORC1	Imatinib mesylate Daunorubicin Focal adhesion kinase (FAK) inhibitor Methotrexate 6-mercaptopurine	NCT01184885 NCT00792948	[80,82,154,155,171]
CCI-779 (Temsirolimus) (Ph^−^ B-ALL)	mTORC1	-	NCT01614197 NCT01403415	[80,81,82,96,144,182]
RAD001 (Everolimus) (Ph^+^ B-ALL, Ph^−^ B-ALL)	mTORC1	Vincristine Bortezomib MK2206 LEE-01 (CDK4/6 inhibitor) Glucocorticoids	NCT01523977	[5,75,80,82,89,96,176,177,178,179,180,181,183]
PI-103 (Ph^+^ B-ALL)	PI3K/mTOR	Imatinib	-	[6,10,77,127,148]
PKI-587 (Gedatolisib) (Ph^+^ B-ALL, Ph^−^ B-ALL, Ph-like B-ALL)	PI3K/mTOR	-	-	[6,76,77,136]
BEZ235 (Ph^+^ B-ALL, Ph^−^ B-ALL)	PI3K/mTOR	Nilotinib GX15-070 Methotrexate 6-mercaptopurine	-	[6,10,84,95,162,181]
BGT226 (Ph^−^ B-ALL)	PI3K/mTOR	-	-	[8,100,101,102]
PP-242 (Ph^+^ B-ALL)	mTORC1/mTORC2	-	-	[80,85,150]
Torin-2 (B-pre-ALL)	mTORC1/mTORC2	MK2206	-	[80,85,180]
MLN0128 (Ph^+^ B-ALL, Ph^−^ B-ALL)	mTORC1/mTORC2	Dasatinib	-	[80,85,164]
